# Expression pattern of cochlear microRNAs in the mammalian auditory hindbrain

**DOI:** 10.1007/s00441-020-03290-x

**Published:** 2020-11-06

**Authors:** Constanze Krohs, Mor Bordeynik-Cohen, Naama Messika-Gold, Ran Elkon, Karen B. Avraham, Hans Gerd Nothwang

**Affiliations:** 1 Neurogenetics Group and Cluster of Excellence Hearing4All, School of Medicine and Health Sciences, Carl Von Ossietzky University Oldenburg, 26111 Oldenburg, Germany; 2 Department of Human Molecular Genetics and Biochemistry, Sackler Faculty of Medicine and Sagol School of Neuroscience, Tel Aviv University, 6997801 Tel Aviv, Israel; 3 Research Center for Neurosensory Science, Carl Von Ossietzky University Oldenburg, 26111 Oldenburg, Germany; 4 Department of Neuroscience, Center of Excellence Hearing4All, Carl Von Ossietzky University Oldenburg, 26111 Oldenburg, Germany

**Keywords:** Development, Gene regulatory network, Auditory system, Auditory rehabilitation, Evolution

## Abstract

The auditory system comprises the auditory periphery, engaged in sound transduction and the central auditory system, implicated in auditory information processing and perception. Recently, evidence mounted that the mammalian peripheral and central auditory systems share a number of genes critical for proper development and function. This bears implication for auditory rehabilitation and evolution of the auditory system. To analyze to which extent microRNAs (miRNAs) belong to genes shared between both systems, we characterize the expression pattern of 12 cochlea-abundant miRNAs in the central auditory system. Quantitative real-time PCR (qRT-PCR) demonstrated expression of all 12 genes in the cochlea, the auditory hindbrain and the non-auditory prefrontal cortex (PFC) at embryonic stage (E)16 and postnatal stages (P)0 and P30. Eleven of them showed differences in expression between tissues and nine between the developmental time points. Hierarchical cluster analysis revealed that the temporal expression pattern in the auditory hindbrain was more similar to the PFC than to the cochlea. Spatiotemporal expression analysis by RNA in situ hybridization demonstrated widespread expression throughout the cochlear nucleus complex (CNC) and the superior olivary complex (SOC) during postnatal development. Altogether, our data indicate that miRNAs represent a relevant class of genetic factors functioning across the auditory system. Given the importance of gene regulatory network (GRN) components for development, physiology and evolution, the 12 miRNAs provide promising entry points to gain insights into their molecular underpinnings in the auditory system.

**Electronic supplementary material:**

The online version of this article (10.1007/s00441-020-03290-x) contains supplementary material, which is available to authorized users.

## Introduction

Hearing requires transduction of weak sound pressure waves in the cochlea and subsequent complex processing of precisely timed electric signals in central auditory structures. To perform these tasks, both the cochlea and the auditory circuits have acquired unique features on the molecular, morphological and cellular levels, such as hair bundles and ribbon synapses in the cochlea (Meyer et al. 2009; Michalski and Petit 2019; Petit and Richardson 2009) and giant high fidelity synapses and ultrafast signaling along the auditory pathways (Carr and Soares 2002; Trussell 1997, 1999; Yu and Goodrich 2014). In therian mammals, the auditory system is further characterized by a unique coiled and elongated cochlea (Manley 2012, 2017) and an unusual high number of processing structures in the hindbrain, such as the composite structures of the cochlear nucleus complex (CNC) and the superior olivary complex (SOC) (Nothwang 2016; Schwartz 1992; Willaredt et al. 2015). Anatomical, developmental and physiological studies led to the conclusion that both the coiled cochlea and the complex composition of the auditory hindbrain represent mammalian-specific traits of the vertebrate auditory system (Carr and Soares 2002; Nothwang 2016; Manley and Clack 2004; Manley 2012).

Despite their disparate functions and unique specializations, the cochlea and the auditory hindbrain share critical genes for proper development and function (Michalski and Petit 2019; Nothwang et al. 2015; Willaredt et al. 2014). The encoded proteins have quite diverse functions, ranging from a role in transcription to axon guidance molecules and proteins involved in neurotransmission. Recent analyses extended the concept of shared genes between the peripheral and central auditory system on the genetic level to non-coding microRNAs (miRNAs) and on the tissue level to the auditory cortex. Mutations in miR-96 affect the development of both the hair cells in the cochlea (Lewis et al. 2009; Mencia et al. 2009) and circuits in the auditory hindbrain (Schlüter et al. 2018) and the essential hair bundle proteins cdhr23 and cdhr15 are also required for proper integration of GABAergic interneurons in the auditory cortex (Libé-Philippot et al. 2017).

The concept of shared critical genes between the peripheral and central auditory system bears implications for both the evolution of the auditory system and auditory rehabilitation (Willaredt et al. 2015, 2014; Michalski and Petit 2019). This warrants a more systematic approach to study the extent and underlying mechanisms of shared genes, especially with a focus on components of gene regulatory networks (GRNs). miRNAs constitute an essential layer of GRNs and are strongly associated with developmental and evolutionary processes (Bartel 2018; Kittelmann and McGregor 2019; Kosik and Nowakowski 2018). Hence, they represent a promising entry point to such studies. miRNAs are short (19–25 nucleotides) non-coding transcripts that regulate gene expression by blocking translation of mRNAs and/or promoting their degradation (Bartel 2018). Their biogenesis begins with transcription of a primary miRNA (pri-miRNA), which is then processed to yield mature miRNAs. Mature miRNAs are incorporated into the RNA-induced silencing complex. This complex subsequently recognizes sequence-specific target sites on mRNAs. This results in translational inhibition or destabilization and subsequent degradation of the targeted mRNA. To explore the miRNA expression pattern between the peripheral and central auditory system, we comprehensively analyzed the expression of 12 miRNAs highly expressed in the sensory epithelium of the mouse cochlea (Rudnicki et al. 2014) and the developing auditory hindbrain. In addition, we included the prefrontal cortex (PFC) in some of our analysis in order to compare expression patterns between auditory and non-auditory structures.

## Materials and methods

### Animals

C57BL/6N mice of both sexes were used at indicated stages for RNA isolation from brain tissue or RNA in situ hybridization experiments. All protocols were approved by the local animal care and use committee (LAVES, Oldenburg). All experiments were in accordance with the regulations of German federal law on the care and use of laboratory animals and followed the guidelines of the EU Directive 2010/63/EU for animal experiments. For isolation of cochlear sensory epithelial RNA, C57BL/6J mice were acquired from Envigo (Jerusalem, Israel) for all developmental time points. All animal procedures were approved by the Animal Care and Use Committee at Tel Aviv University (01-17-098) and adhered to guidelines set forth by the National Institutes of Health Guide for the Care and Use of Laboratory Animals.

### Quantitative real-time PCR

In order to isolate RNA from postnatal brain tissue, mice were sacrificed with CO_2_ and decapitated at postnatal day (P)0 or P30. The brain was immediately prepared out of the skull and frozen on dry ice. For 16-day-old embryos (E16), caesarian sections were performed on timed pregnant animals. Embryos were decapitated and their heads frozen on dry ice. The SOC or the PFC, respectively, were cut out from 300-µm-thick coronal sections, stored in RNAlater (QIAGEN, Hilden, Germany) and the tissue of several animals was pooled for RNA isolation. For the PFC, attention was paid to exclude tissue from the auditory cortex, as it served as a non-auditory structure of the central nervous system. The tissue of 8 individuals per sample was pooled for E16 animal and 6–8 individuals per P0 sample and 3 individuals per P30 sample were collected. Total and small RNA was isolated with the innuPrep miRNA Kit (Analytik Jena, Jena, Germany) and frozen at − 80 °C. The RNA concentration was determined with a Nanophotometer (Implen GmbH, München, Germany) and RNA integrity was controlled with a 2000 Bioanalyzer (Agilent, CA, USA).

For RNA from cochlear tissue, the inner ears of 6–8 mice at E16, P0, or P30 were dissected to create a pool of cochlear sensory epithelium, as previously described (Rudnicki et al. 2014). Epithelia was dissected directly into RNAlater solution. For P30 only, tissue was incubated in RNAlater for 8–10 h to allow full penetration prior extraction. To extract total RNA, Direct-zol™ RNA Miniprep (Zymo Research, CA, USA) was used, applying a 23G blunt end needle (Instech, PA, USA) to shear the tissue while incubating with TRI Reagent (Zymo Research, CA, USA). Finally, RNA quality was assessed using a NanoDrop 2000 spectrophotometer (ThermoFisher Scientific, MA, USA) and TapeStation 4200 (Agilent, CA, USA) and stored at − 80 °C.

qScript cDNA Synthesis Kit (Quanta Biosciences, MA, USA) was used to reverse transcribe total RNA. Each 10 μl reaction contained 3 μl of total RNA diluted to 10 ng/μl, 2 μl of cDNA mix and 1.5 μl of RT-primer. RT-primers and probes (TM-primers) were ordered as TaqMan microRNA Assays (Applied Biosystems, CA, USA) for miR-26a-5p, miR-204-5p, miR-27b-3p, miR-127-3p, miR-22-3p, miR-183-5p, miR-181c-5p, miR-143-3p, let-7c-5p, miR-191-5p (Assay ID. 000405, 000508, 000409, 000452, 000398, 002269, 000482, 002249, 000379 and 002299, respectively). RT primers and probes for miR-181a-5p, miR-181b-5p and U6-snRNA (Applied Biosystems, CA, USA) were a kind gift from Prof. Carmit Levy (Assay ID. 000480, 478583 and 001093, respectively). U6-snRNA was used as internal reference gene for normalization of expression levels, i.e., to correct for differences in absolute mRNA content, sample preparation, etc. No template was used as a negative control. Each 10 μl quantitative real-time PCR (qRT-PCR) reaction contained 5 μl of TaqMan mix FastStart Universal Probe Master with Rox (Roche, Switzerland), 0.5 μl of TM-primer and 1 μl of cDNA. qPCR was performed on a QuantStudio 12K Flex Real-Time PCR System (Applied Biosystems). All qRT-PCRs were performed in three biological repeats, each in triplicates. miRNA expression analysis was based on a standard two-way ANOVA test, followed by Tukey’s post hoc test. Significance values were set to *P* < 0.05. *P* values for the main effects (that is, developmental time point and tissue) were adjusted for multiple testing (*n* = 12 miRs) using BH FDR correction (Benjamini and Hochberg 1995). Similarity relationships between miRs over the probed conditions were outlined by hierarchical clustering of the *∆Ct* values (after averaging over replicate samples) using Euclidean distance. All statistical analyses were carried out in R.

### RNA in situ hybridization (qualitative analysis of miRNA expression)

Digoxigenin-labeled RNA-probes against the pri-miRNAs mmu-miR-22, mmu-miR-26a, mmu-miR-27b, mmu-miR-127, mmu-miR-143, mmu-miR-181a, mmu-miR-181b, mmu-miR-181c, mmu-miR-183, mmu-miR-191, mmu-miR-204 and mmu-miR-let-7c were generated as follows: PCR products of every pri-miRNA were cloned into the pGEMT-Easy vector (Promega, Madison, Wisconsin, USA). For accession numbers of microRNAs and PCR primers, see Table 1. In vitro transcription of sequence verified clones with the T7 polymerase in the presence of digoxigenin-11-UTP (Roche Applied Science) resulted in antisense probes encompassing the precursors and partial primary transcripts of miRNAs. Due to the double-stranded hairpin structure of pri-miRNAs, sense probes for pri-miRNAs show partial complementarity to the target and therefore hybridize as well. We therefore used only antisense probes. Specificity of the probes was indicated by their different expression patterns. Furthermore, more than 40 sense probes in previous studies using the same protocol provided negative results (Ehmann et al. 2013; Pawlik et al. 2016).

**Table 1:** Accession numbers and primers for the generation of RNA probes

**MicroRNA**	**MirBase Accession**	**NCBI Accession**	**Forward Primer**	**Reverse Primer**
**mmu-miR-22**	MI0000570	NC_000077.6	5´-GCCAGTTGAAGAACTGTTGCC-3´	5´-AGACCTTCCCACCCCAGTT-3´
**mmu-miR-26a-1**	MI0000573	NC_000075.6	5´-CAAAAGCTGGAGGACCGAGG-3´	5´-GGAAACTCTGTTGTTGCCGC-3´
**mmu-miR-27b**	MI0000142	NC_000079.6	5´-AGCCTTCGAAGATGCTCACC-3´	5´-TCTCCTCCTCTGGAGTGACC-3´
**mmu-miR-127**	MI0000154	NC_000078.6	5´-TTGCTGCCTGGCTTTCTCTT-3´	5´-CATACTCAGACCTGGCCGAC-3´
**mmu-miR-143**	MI0000257	NC_000084.6	5´-AGACCCGGATAGGAGGCAG-3´	5´-CCAACACTTACCACGTCCCG-3´
**mmu-miR-181a-1**	MI0000697	NC_000067.6	5´-ATCTCTGCCTCACAGGTTGC-3´	5´-CTGAAGAGGCGGGGAGAATC-3
**mmu-miR-181b-1**	MI0000723	NC_000067.6	5´-TGAAGACAGAACCGCAAAGC-3´	5´-GATTGCGACAGCAAAAAGCG-3´
**mmu-miR-181c**	MI0000724	NC_000074.6	5´-CCCTGGTTTCTCTCTCGTCC-3´	5´-GGTCTACAGGGTGGGGATGG-3´
**mmu-miR-183**	MI0000225	NC_000072.6	5´-TGGAGAGTGTGACTCCTGTC-3´	5´-GTCTAGGCAGAAAGGGGTGAG-3´
**mmu-miR-191**	MI0000233	NC_000075.6	5´-TCCTTCCTACTCAGCCCACT-3´	5´-AAGTGCAGCTGGAATGCTCT-3´
**mmu-miR-204**	MI0000247	NC_000085.6	5´-GCAGGAAATGAAGAGGTTGGC-3´	5´-TCCACGAGTCACATGAAGAAGG-3´
**mmu-let-7c-1**	MI0000559	NC_000082.6	5´-TCTACAACCTTGCCAAGCCC-3´	5´-GATGGCTCAAGTGTGCTCCA-3´

Mice were injected intraperitoneally with a lethal dose of sodium pentobarbital (Narcoren©, Merial, Lyon, France; 650 mg/kg bodyweight) and perfused transcardially with phosphate-buffered saline (PBS, 136.9 mM NaCl, 2.7 mM KCl, 10.1 mM Na_2_HPO_4_, 1.8 mM KH_2_PO_4_, pH 7.4) followed by 4% PFA (4% paraformaldehyde in PBS, pH 7.4). Brains were postfixed in 4% PFA overnight and incubated for at least 16 h in 30% sucrose in PBS. Brains were embedded in Tissue Freezing Medium (TBS, Durham, NC, USA) and stored at − 80 °C until analysis. Coronal sections of 20 µm thickness were cut on a cryostat (Leica Biosystems, Nußloch, Germany) and stored at − 80 °C.

On-slide in situ hybridization was performed as follows. Slices were treated with proteinase K (10 µg/ml) for 8 min and deacetylated for 10 min (12.5 µl acetic anhydride in 5 ml 0.1 M triethanolamine in 0.9% NaCl). Slices were then incubated 2 h at 50 °C in hybridization buffer (50% formamide, 5× SSC (saline sodium citrate, prepared from a stock of 20× SSC (3 M NaCl, 300 mM Na_3_-citrate, pH 7.0)), 2% blocker (Roche Applied Science, Penzberg, Germany), 0.02% SDS, 0.1% N-lauryl-sarcosine), followed by an overnight incubation at 50 °C in hybridization buffer containing 1 µg/ml RNA probe. After washing for 30 min each at 45 °C with 2× SSC, 0.5× SSC and PBS 1% Tween, slices were incubated for 1 h with blocking solution (1% blocking reagent (Roche Applied Science)) in maleic acid buffer (0.1 M maleic acid, 0.15 M NaCl, pH 7.5) at room temperature (RT) followed up by a 1.5-h incubation with an alkaline phosphatase conjugated antibody against digoxigenin (Anti-DIG AP, Roche Applied Science) in a 1:1000 dilution in blocking solution. Signal detection was performed in the presence of 5-bromo, 4-chloro, 3-indolylphosphate (BCIP)/nitro-blue tetrazolium chloride (NBT), Roche Applied Science) 1:50 in AP-Buffer (100 mM Tris,150 mM NaCl, 5 mM MgCl_2_, pH 9.5) at RT. Results were documented with an AxioScan Z1 (Zeiss, Oberkochen, Germany). In situ hybridization was repeated at least three times for each probe on at least three different animals. Images shown are representative results.

## Results

### qRT-PCR based expression analysis during development

To approximate the extent of shared expression of miRNAs between the peripheral and central auditory system, we selected 12 highly expressed miRNAs from an unbiased miRNA-seq approach in the sensory epithelium including hair cells, supporting cells, etc. of the mouse cochlea at P0 (Rudnicki et al. 2014). These were miR-22, miR-26a, miR-27b, miR-127, miR-143, miR-181a, miR-181b, miR-181c, miR-183, miR-191, miR-204 and miR-let-7c. In a first set of experiments, we performed qRT-PCR to study the expression of the mature forms in more detail during development in both the cochlea and the SOC, a prominent composite auditory hindbrain structure. For comparison, we included the prefrontal cortex (PFC) as a non-auditory structure of the central nervous system. As miRNAs play an important role during development, we chose the E16, P0 time points that cover embryonic and perinatal stages characterized by ongoing highly dynamic developmental processes and P30, a time point when the auditory system is fully functional.

**Fig. 1:**
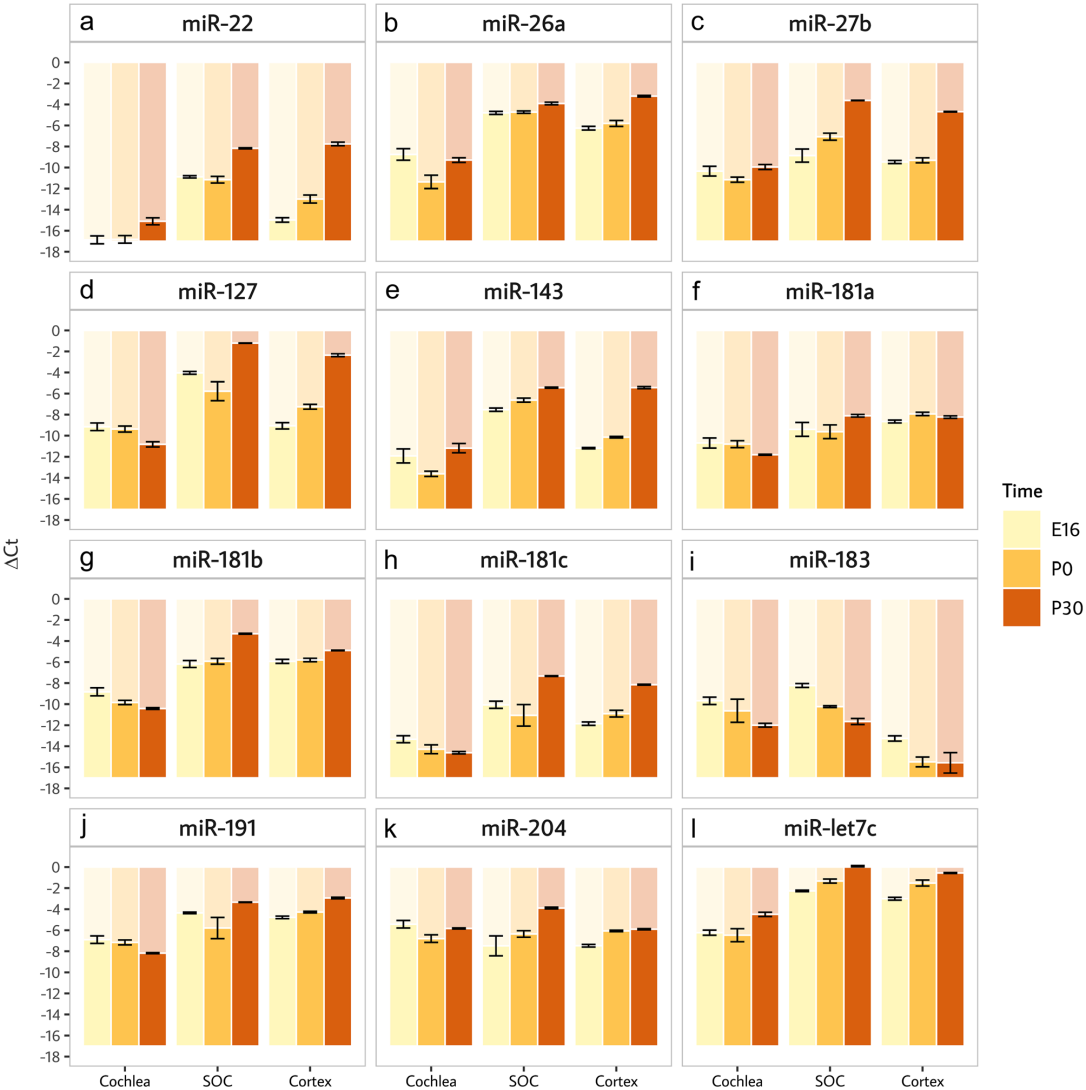
miRNAs expressed at all stages in the cochlea, SOC and cortex. Bar plots for *∆Ct* values measured for each miRNA across the three tissues and developmental time points (E16, P0 and P30). Figure parts a– l correspond to the expression of indicated miRNAs. Levels are presented as means (over independent triplicates) ± SE

All 12 miRNAs were expressed at all stages in all three tissues, i.e., the cochlea, the SOC and the neocortex (Fig. 1). Eleven out of the twelve miRNAs (all but miR-204) showed significant (FDR = 5%) difference in expression between tissues (Suppl. Table 1). Three different patterns were observed. Seven miRNAs showed higher expression in both nervous tissues compared to the cochlea (miR-26a, miR-27b, miR-181a, miR-181b, miR-181c, miR-191 and let7c). In all these cases, expression was higher in the nervous system compared to the cochlea (Fig. 1a, b, f, g, h, j, l). Three miRNAs showed significant differences between all three tissues (miR-22, miR-127 and miR-143 (Fig. 1a, d, e). Finally, miR-183 showed significant differences in expression between the two auditory tissues, i.e., cochlea and SOC, compared to the cortex (Fig. 1i), in agreement with its denomination as a neurosensory miRNA (Dambal et al. 2015). Nine miRNAs showed significant differential expression (FDR = 5%) over the developmental time points. Post hoc tests indicated that eight miRNAs are differentially expressed between P0 and P30 (miR-22, miR-26a, miR-27b, miR-127, miR-143, miR-181b, miR-181c and let-7c) and seven miRNAs between E16 and P30 (miR-22, miR-27b, miR-127, miR-143, miR-183, miR-204, let7c) (Suppl. Table 1). Most of these miRNAs showed increased expression over time. A notable exception was miR-183 with down-regulation between E16 and P30 in all three tissues analyzed (Fig. 1i). Interestingly, miR-127 and miR-181b showed up-regulation in the nervous system but down-regulation in the cochlea (Fig. 1d, g). miR-22 and miR-127 were the only miRNAs which showed for a given time point significant differences in expression between the SOC and non-SOC tissues (Fig. 1a, d). At E16, they were more highly expressed in the SOC compared to the cochlea and the cortex.

**Fig. 2:**
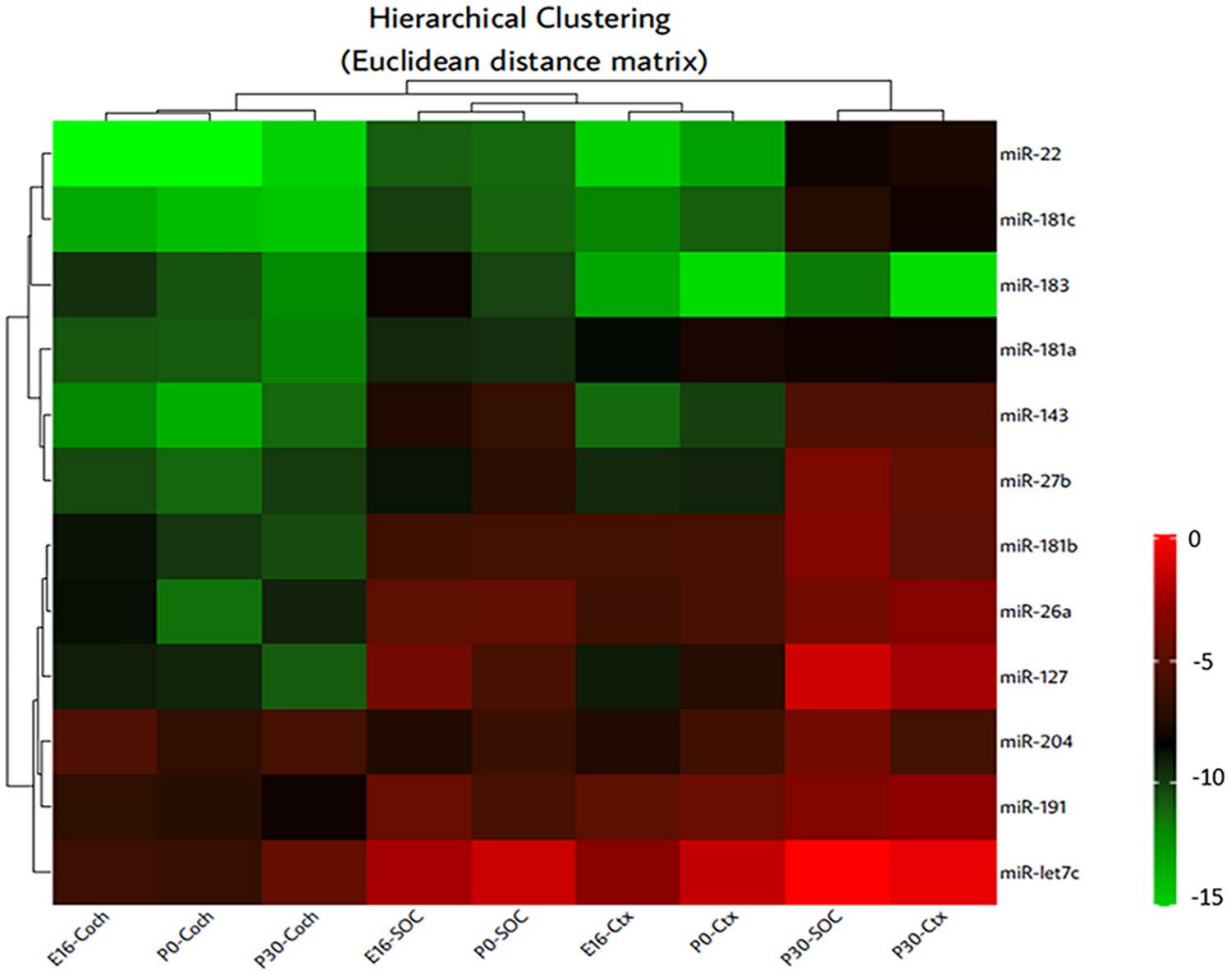
Expression patterns in the SOC and PFC compared to the cochlea. Hierarchical clustering was applied to the twelve miRs over nine biological conditions (tissues and developmental time points) based on *∆Ct* values

Visual inspection indicated a closer relationship between the expression patterns in the SOC and PFC compared to the cochlea. This was mainly due to the developmental upregulation of several miRNAs in the nervous system, whereas in the cochlea, most miRNAs showed a developmental decrease in expression. Hierarchical cluster analysis confirmed the closer relationship of the nervous system structures (Fig. 2). The analysis grouped the cochlea at all three developmental time points separate from the central nervous system tissues. The adult SOC and PFC showed also a strong correlation in expression, whereas during development, the tissue origin (SOC or PFC) determined the clustering. This might reflect differences in timing origin of the developmental processes between the hindbrain and cortical tissue (Caviness 1982; Willaredt et al. 2015; Rice and van der Loos 1977). Thus, all 12 miRNAs analyzed demonstrate expression in both the cochlea and the auditory hindbrain.

### Spatiotemporal expression analysis in the CNC and SOC by RNA in situ hybridization

The qRT-PCR experiments provided insight into the temporal expression pattern of the miRNAs but lacked information on the spatial expression within the auditory hindbrain. The CNC, for instance, is composed of the dorsal cochlear nucleus (DCN) and the anterior and posterior ventral nuclei (aVCN and pVCN) and the murine SOC is mainly made up by the lateral superior olive (LSO), the medial, lateral and ventral nuclei of the trapezoid body (MNTB, LNTB, VNTB and the superior paraolivary nucleus (SPN). To gain insight into the spatiotemporal expression pattern within these nuclei, we performed RNA in situ hybridization. As initial experiments with locked nucleic acids (LNAs) for the mature miRNAs provided poor results, we generated probes against pri-miRNAs. RNA in situ hybridization was then performed on P4 and P30-old tissue sections, as especially the individual nuclei of the SOC become only clearly discernible at P4 onwards (Ebbers et al. 2015). Furthermore, most miRNAs showed higher expression at later stages.

**Fig. 3:**
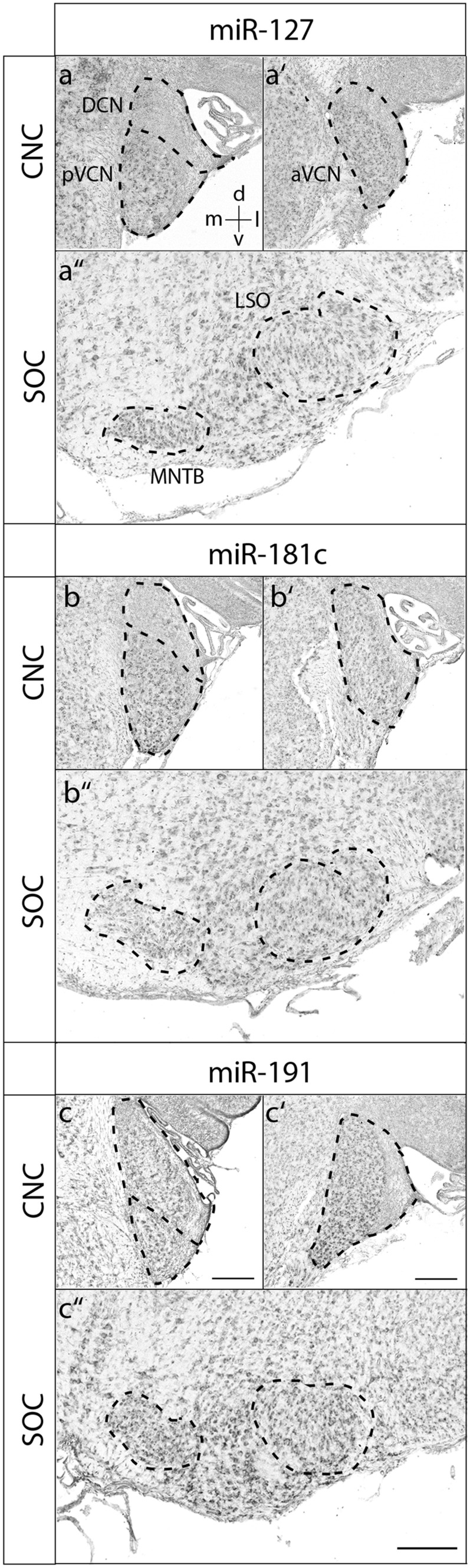
Expression of miR-127, miR-181c and miR-191 at P4. RNA in situ hybridization of coronal sections through the mouse auditory brainstem of P4. The orientation of tissue sections within the brain is indicated by a coordinate system in the upper left image, which applies to all images of the panel. Sections were hybridized with DIG-labeled RNA antisense probes. The dashed lines indicate the positions of respective auditory nuclei. aVCN anterior ventral cochlear nucleus, CNC cochlear nucleus complex, d dorsal, DCN dorsal cochlear nucleus, l lateral, LSO lateral superior olive, m medial, MNTB medial nucleus of the trapezoid body, pVCN posterior ventral cochlear nucleus, SOC superior olivary complex, v ventral. Representative results from at least 3 independent experiments are shown. Scale bars 200 µm; also applies to Figs. 4-6.

**Fig. 4:**
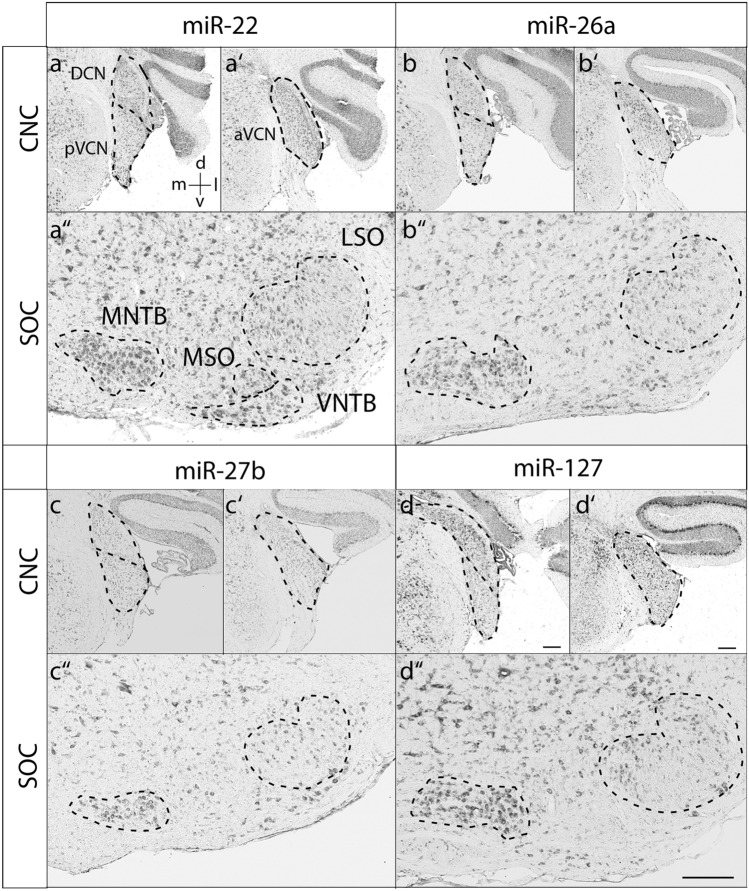
Expression of miR-22, miR-26a, miR-27b and miR-127 at P30. RNA in situ hybridization of coronal sections through the mouse auditory brainstem of P30 animals. MSO medial superior olive, VNTB ventral nucleus of the trapezoid body

**Fig. 5:**
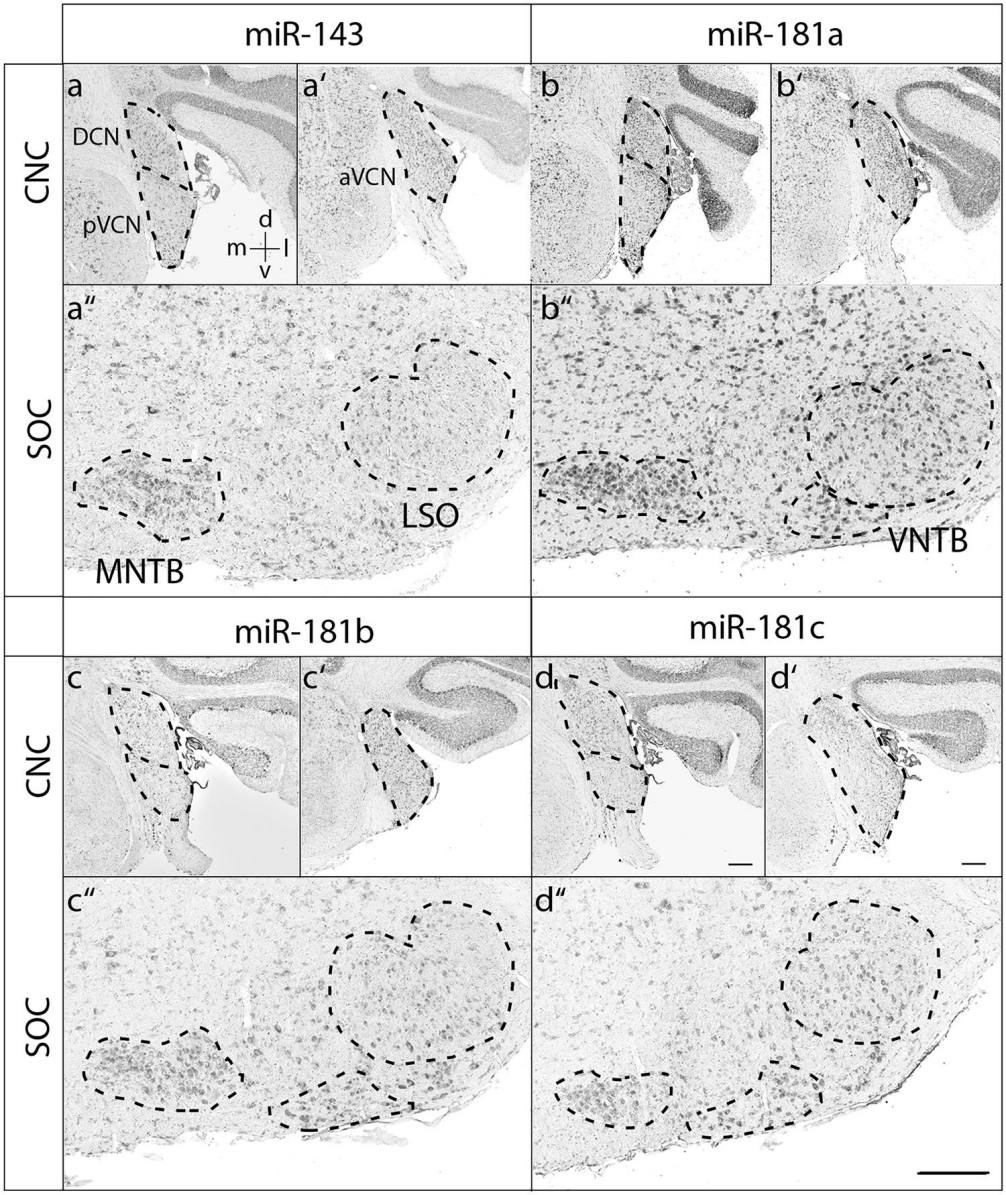
Expression of miR-143, miR-181a, mir-181b and miR-181c at P30. RNA in situ hybridization of coronal sections through the mouse auditory brainstem of P30 animals

**Fig. 6:**
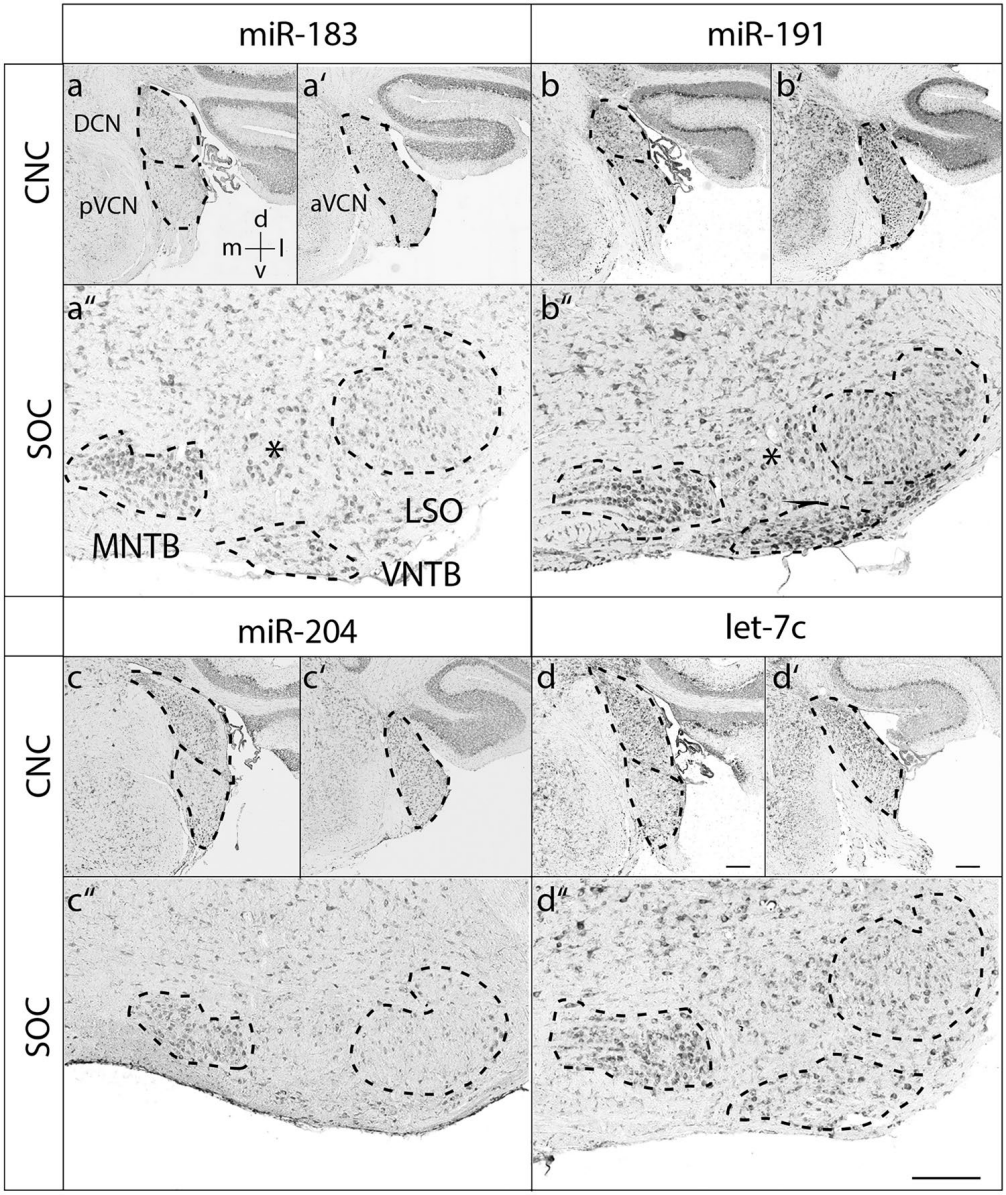
Expression of miR-183, miR-191, miR-204 and let-7c at P30. RNA in situ hybridization of coronal sections through the mouse auditory brainstem of P30 animals

At P4, most miRNAs were broadly expressed within both the CNC and SOC (Fig. 3 and Suppl. Figs. 1–3). Notable exceptions were mir-127 and mir-181c, as the DCN was devoid of any detectable signals for these two miRNAs (Fig. 3). Furthermore, mir-191 showed a tonotopic gradient with high expression in the ventral, low-frequency area of aVCN (Fig. 3). At P30, the expression pattern was more differentiated for most of the miRNAs and the signals in general lower. mir-22 was expressed throughout the CNC. In the SOC, the MNTB and the VNTB showed very prominent signals (Fig. 4). Expression of miR-27b became low throughout the CNC and was strongest in MNTB and large neurons of the LNTB (ventral to the LSO). A notable developmental change was observed for mir-127 and mir-181c, as their expression pattern shifted from pVCN to DCN between P4 and P30 (Figs. 4 and 5). In addition, mir-127 gave very strong signals in the MNTB and labeled in the LSO mainly dorsally located neurons (Fig. 4). mir-191 was intense throughout the CNC and SOC, where prominent signals were also present in the VNTB and LNTB. Remarkably, the gradient in aVCN seemed to be inversed compared to P4 with high expression in the dorsal, high-frequency area (Fig. 6). The remainder miRNAs, mir-26a, mir-143, mir-181a, mir-181b, mir-183, mir-204 and let7c, were rather uniformly expressed throughout the CNC and SOC (Figs. 4-6).

In summary, all 12 precursor miRNAs were expressed in both the CNC and SOC in the prehearing stage (P4) and after hearing-onset (P30).

## Discussion

Here, for the first time, we compared systematically the expression pattern of GRN components between the peripheral and central auditory system. All 12 cochlear miRNAs were expressed in the auditory hindbrain at all stages analyzed. Although limited in scope, these results indicate a considerable overlap between GRN components of the peripheral and central auditory system. Whether this reflects an exceptional developmental and/or evolutionary link warrants further, more comprehensive and detailed studies.

In our analysis, we focused on miRNAs as they are essential components of GRNs during development and thought to play an important role in the evolution of development and diversification of animals (Tarver et al. 2013; Kittelmann and McGregor 2019), including brain function (Somel et al. 2011). miRNAs might hence be of relevance to the mammalian auditory system, as both the cochlea and auditory hindbrain structures such as the CNC and SOC represent mammalian-specific traits (Manley 2012, 2017; Nothwang 2016). Furthermore, transcription factors associated with hearing impairment are enriched in the transcriptome of the SOC (Ehmann et al. 2013) and an interplay between miRNAs and TFs in mixed regulatory circuits was proposed as a building block in regulatory networks underlying developmental genetic programs (He and Hannon 2004; Hornstein and Shomron 2006; Hobert 2008).

We selected miRNAs that are abundantly expressed in the cochlear sensory epithelium at P0. For two of them, a functional role in the peripheral auditory system has been established. miR-183 is implicated in cell fate decisions in the cochlea (Zhou et al. 2018; Li et al. 2010) and miR-204 suppresses cochlear spiral ganglion neuron survival (Li et al. 2014). For the other miRNAs, nothing is known about their function in the inner ear (Zhou et al. 2018; Li et al. 2010, 2014). Their high expression was taken as an indicator for an important role in the inner ear. We renounced further selection criteria such as predicted target genes, established roles in neuronal development (Kosik and Nowakowski 2018; Schratt 2009), or evolutionary conservation (Bartel 2018; Berezikov 2011) in order to avoid strong biases in our analysis.

What might be the function of the analyzed miRNAs in the auditory hindbrain? For miR-183, one might hypothesize a function similar to miR-96. Both miRNAs belong to the miRNA cluster miR-183, miR-96 and miR-182, which is transcribed as a polycistronic transcript. Indeed, miR-183 showed a similar expression pattern as miR-96 in the auditory hindbrain (Pawlik et al. 2016). A mutation in miR-96 causes reduced cell size, altered electrophysiological properties, changes in gene expression and impaired maturation of a giant synapse in the auditory hindbrain (Schlüter et al. 2018). Both miRNAs share the identical seed region (Dambal et al. 2015), which is involved in target recognition. This shared property, together with their similar expression pattern, makes it likely that both miRNAs cooperate in their function. The other miRNAs have also been linked to functions in the nervous system, such as neuronal differentiation (miR-143), neurite outgrowth (miR-26a, miR-127, miR-181a, miR-181b, miR-181c), neuronal cell death (miR-22, miR-204), synaptogenesis and neurotransmission (miR-27b), synaptic plasticity (miR-191), or neuroprotection (miR-22, let-7c). Since the action of miRNAs is dependent on the mRNA-transcriptome, functional data from other tissues or organs offer only limited amount of information. Furthermore, most of them showed highest expression at P30, suggesting a role in the mature auditory system. Determination of their precise role in the auditory hindbrain therefore requires an analysis in animals with targeted deletion of the respective gene, most advantageously confined to the central nervous system. miR-127 and miR-181c represent attractive candidates for such an approach. They show a shift of expression from the pVCN at P4 to the DCN at P30, have been involved in regulation of neurite outgrowth and are the only two miRNAs among the twelve that are specific to eutherians (Bartel 2018).

In general, expression at P30 appeared lower than at P4, both in number of labeled cells and intensity of the signals. This contrasted the developmental up-regulation of many miRNAs as demonstrated by qRT-PCR. The most likely explanation for this discrepancy is that the qRT-PCR quantified mature miRNAs, whereas the RNA in situ hybridization probes were directed against the pri-miRNAs. Indeed, posttranscriptional regulation of the miRNA biogenesis was already shown to result in considerable differences between the expression level of the precursor miRNA and its mature form (Siomi and Siomi 2010; Obernosterer et al. 2006). For example, during development, numerous pri-miRNAs are expressed but are not efficiently converted into mature miRNAs (Thomson et al. 2006). The apparently high expression of pri-miRNAs at P4 in the auditory hindbrain is therefore compatible with a low expression of the respective mature forms.

In summary, all twelve cochlear miRNAs analyzed in this study are expressed in the central auditory system as well. This indicates that miRNAs are attractive candidates for genes critically involved in development and function of the auditory system. Consequently, any mutation in miRNAs associated with impaired hearing requires likely functional analysis in both the peripheral and central auditory system to estimate the benefit of auditory rehabilitation by hearing devices. Given that changes in the expression of GRNs underlie many examples of phenotypic evolution (Carroll 2008; Peter and Davidson 2011, 2015; Heimberg et al. 2008), comparative analysis of miRNA expression in the vertebrate auditory system will likely shed light on molecular mechanisms underlying evolutionary development of the mammalian auditory system.

## Electronic supplementary material

Below is the link to the electronic supplementary material.

Supplementary file1 (TIF 3241 kb)

Supplementary file2 (TIF 3205 kb)

Supplementary file3 (TIF 3218 kb)

Supplementary file4 (XLSX 27 kb)
