# Supplementary figures and images for: Expression pattern of cochlear microRNAs in the mammalian auditory hindbrain
Source: Cell Tissue Res. 2020 Nov 6;383(2):655–66. doi: 10.1007/s00441-020-03290-x (PMC7904729; doi:10.1007/s00441-020-03290-x)

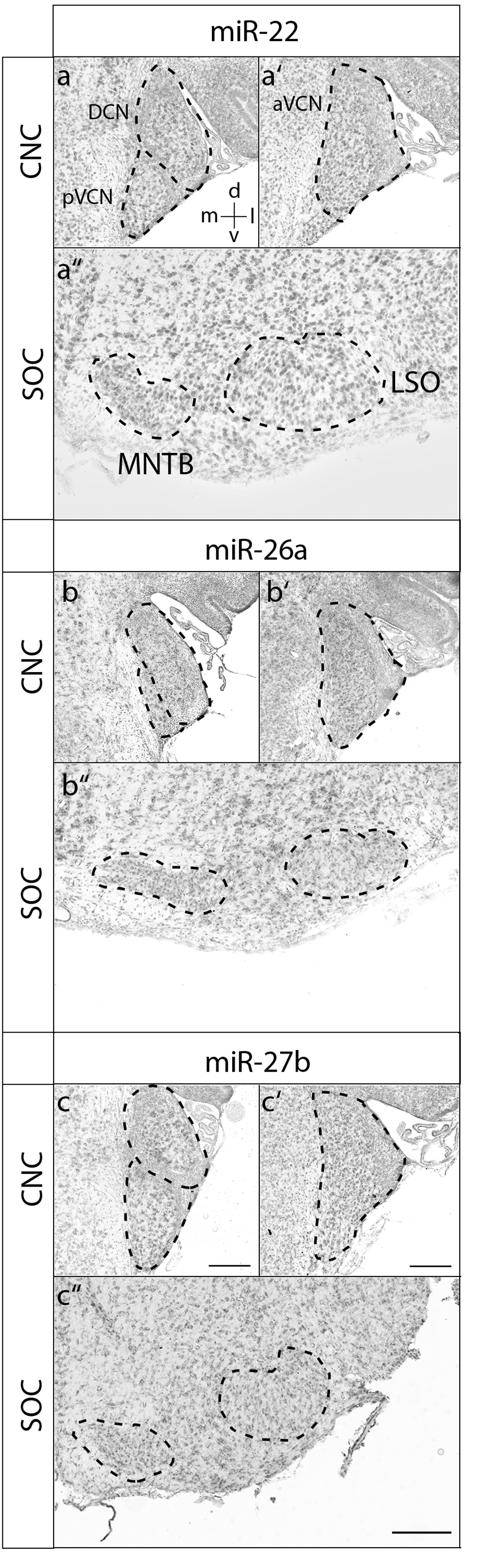

Supplement: Supplementary file 1 — Supplementary file1 (TIF 3241 kb) [file 441_2020_3290_MOESM1_ESM.tif]

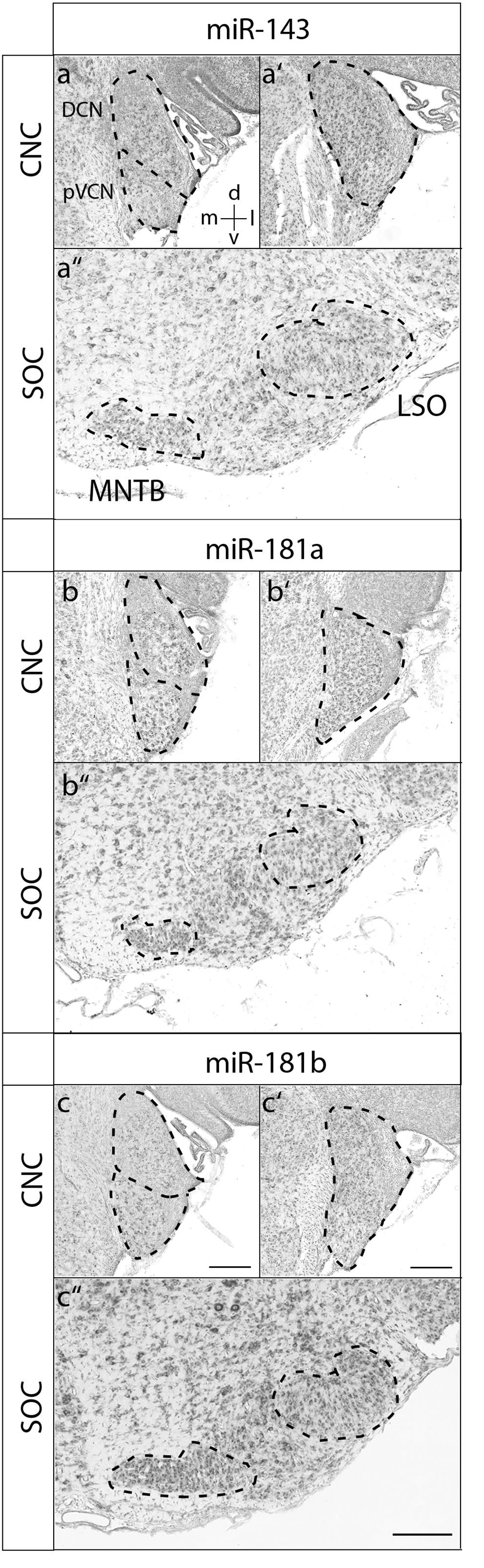

Supplement: Supplementary file 2 — Supplementary file2 (TIF 3205 kb) [file 441_2020_3290_MOESM2_ESM.tif]

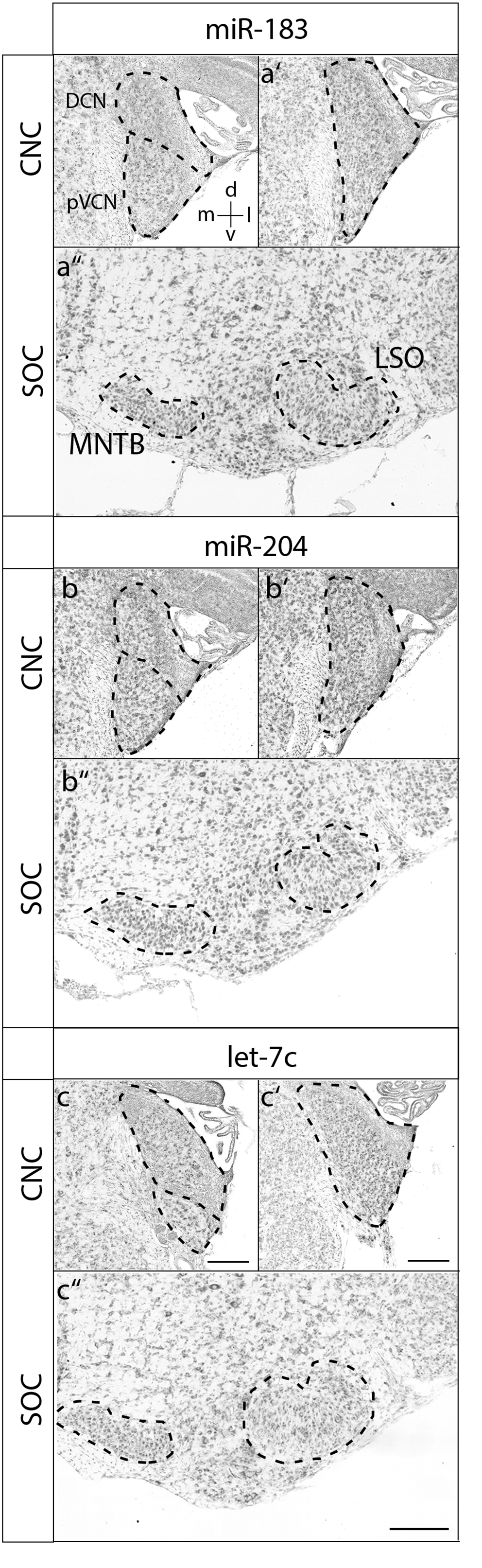

Supplement: Supplementary file 3 — Supplementary file3 (TIF 3218 kb) [file 441_2020_3290_MOESM3_ESM.tif]
